# Supplementary material for: Mechanistic Signatures of Human Papillomavirus Insertions in Anal Squamous Cell Carcinomas
Source: Cancers (Basel). 2019 Nov 22;11(12):1846. doi: 10.3390/cancers11121846 (PMC6966520; doi:10.3390/cancers11121846)
Supplement: Supplementary file 1 [file cancers-11-01846-s001.pdf]

Supplementary Table S1. Clinical and biological characteristics of the 93 patients with HPV-positive anal squamous cell carcinoma (ASCC), according to HPV viral load.

	Patients (%)	Number of patient (%)		*p*-value[a]
		Low HPV load (<12)	High HPV load (≥12)	
Total	93 (100)	41 (44.1)	52 (55.9)	
Age (years)				
<65	60 (64.5)	26 (63.4)	34 (65.4)	0.84 (NS)
≥65	33 (35.5)	15 (36.6)	18 (34.6)	
Gender				
Male	18 (19.4)	33 (80.5)	42 (80.8)	0.97 (NS)
Female	75 (80.6)	8 (19.5)	10 (19.2)	
Tumor differentiation				
Well/Moderate	83 (89.2)	37 (90.2)	46 (88.5)	0.95 (NS)
Poor	10 (10.8)	4 (9.8)	6 (11.5)	
Basaloid contingent				
Present	9 (9.7)	2 (4.9)	7 (13.5)	0.30 (NS)
Absent	84 (90.3)	39 (95.1)	45 (86.5)	
HPV status				
Genotype 16	85 (91.4)	36 (87.8)	49 (94.2)	0.12 (NS)
Other genotypes	8 (8.6)	5 (12.2)	3 (5.8)	
Concomitant HIV infection [b]				
Yes	8 (8.8)	5 (12.8)	3 (5.8)	0.14 (NS)
No	83 (91.2)	34 (87.2)	49 (94.2)	
Tumor stage (AJCC 2010) [c]				
I	8 (8.9)	4 (10.3)	4 (7.8)	0.98 (NS)
II	37 (41.1)	15 (38.5)	22 (43.1)	
IIIA	21 (23.3)	9 (23.1)	12 (23.5)	
IIIB	20 (22.2)	9 (23.1)	11 (21.6)	
IV	4 (4.4)	2 (5.1)	2 (3.9)	
Lymph node invasion [b]				
Yes	40 (44.0)	17 (43.6)	23 (44.2)	0.95 (NS)
No	51 (56.0)	22 (56.4)	29 (55.8)	
Synchronous metastasis [d]				
Yes	4 (4.3)	2 (5.0)	2 (3.8)	0.81 (NS)
No	88 (95.7)	38 (95.0)	50 (96.2)	
Sample status				
Treatment-naive tumor	54 (58.1)	20 (48.8)	34 (65.4)	0.11 (NS)
Tumor recurrence	39 (41.9)	21 (51.2)	18 (34.6)	
Complete Response after RT/CRT [e]				
Yes	72 (86.7)	31 (91.2)	41 (83.7)	0.51 (NS)
No	11 (13.3)	3 (8.8)	8 (16.3)	
PIK3CA mutational status				
Wild-type	71 (76.3)	31 (75.6)	40 (76.9)	0.88 (NS)
Mutated	22 (23.7)	10 (24.4)	12 (23.1)	

[a] Chi-Square Test, Chi-Square Test with Yates' correction or Fisher's exact Test, as appropriate; *p* values for comparison of the Low HPV load group vs. the High HPV load group for each parameter
[b] Data available for 91 patients

[c] Data available for 90 patients
[d] Data available for 92 patients
[e] Data available for 83 patients
CRT: chemo-radiotherapy; RT: radiotherapy

Supplementary Table S2. Association between mechanisms of integration of HPV and clinical, biological and pathological characteristics of the 93 patients with HPV-positive anal squamous cell carcinoma (ASCC).

	Patients (%)	Number of patients (%)						*p*-value[a]
		EPI	*2J-COL*	*2J-NL*	*MJ-CL*	*MJ-SC*	*Other*	
Total	93 (100)	42 (45.2)	7 (7.5)	2 (2.2)	9 (9.7)	25 (26.9)	8 (8.6)	
Age (years)								
<65	60 (64.5)	28 (66.7)	5 (71.4)	1 (50.0)	7 (77.8)	13 (52.0)	6 (75.0)	0.67 (NS)
≥65	33 (35.5)	14 (33.3)	2 (28.6)	1 (50.0)	2 (22.2)	12 (48.0)	2 (25.0)	
Gender								
Male	18 (19.4)	7 (16.7)	1 (14.3)	0 (0.0)	2 (22.2)	6 (24.0)	2 (25.0)	0.93 (NS)
Female	75 (80.6)	35 (83.3)	6 (85.7)	2 (100.0)	7 (77.8)	19 (76.0)	6 (75.0)	
Tumor differentiation								
Well/Moderate	83 (89.2)	37 (88.1)	6 (85.7)	2 (100.0)	9 (100.0)	21 (84.0)	8 (100.0)	0.68 (NS)
Poor	10 (10.8)	5 (11.9)	1 (14.3)	0 (0.0)	0 (0.0)	4 (16.0)	0 (0.0)	
Basaloid contingent								
Present	9 (9.7)	6 (14.3)	0 (0.0)	0 (0.0)	0 (0.0)	3 (12.0)	0 (0.0)	0.56 (NS)
Absent	84 (90.3)	36 (85.7)	7 (100.0)	2 (100.0)	9 (100.0)	22 (88.0)	8 (100.0)	
HPV status								
Genotype 16	85 (91.4)	36 (85.7)	6 (85.7)	2 (100.0)	9 (100.0)	24 (96.0)	8 (100.0)	0.96 (NS)
Other genotypes	8 (8.4)	6 (14.3)	1 (14.3)	0 (0.0)	0 (0.0)	1 (4.0)	0 (0.0)	
Concomitant HIV infection [b]								
Yes	8 (8.8)	3 (7.3)	1 (14.3)	0 (0.0)	0 (0.0)	3 (12.5)	1 (12.5)	0.98 (NS)
No	83 (91.2)	38 (92.7)	5 (85.7)	2 (100.0)	9 (100.0)	21 (87.5)	7 (87.5)	
Tumor stage (AJCC 2010) [c]								
I	8 (8.9)	5 (11.9)	1 (14.3)	0 (0.0)	1 (11.1)	1 (4.5)	0 (0.0)	0.95 (NS)
II	37 (41.1)	18 (42.9)	3 (42.9)	1 (50.0)	2 (22.2)	9 (40.9)	4 (50.0)	
IIIA	21 (23.3)	9 (21.4)	0 (0.0)	1 (50.0)	3 (33.3)	6 (27.3)	2 (25.0)	
IIIB	20 (22.2)	8 (19.0)	2 (28.6)	0 (0.0)	2 (22.2)	6 (27.3)	2 (25.0)	
IV	4 (4.4)	2 (4.8)	1 (14.3)	0 (0.0)	1 (11.1)	0 (0.0)	0 (0.0)	
Lymph node invasion [b]								
Yes	40 (44.0)	14 (33.3)	3 (42.9)	1 (50.0)	6 (66.7)	12 (48.0)	4 (50.0)	0.47 (NS)
No	51 (56.0)	28 (66.7)	4 (57.1)	1 (50.0)	3 (33.3)	11 (44.0)	4 (50.0)	
Synchronous metastasis [d]								
Yes	4 (4.3)	2 (4.8)	1 (14.3)	0 (0.0)	1 (11.1)	0 (0.0)	0 (0.0)	0.52 (NS)
No	88 (95.7)	40 (95.2)	6 (85.7)	2 (100.0)	8 (88.9)	24 (100.0)	8 (100.0)	
Sample status								
Treatment-naive tumor	54 (58.1)	25 (59.5)	4 (57.1)	1 (50.0)	4 (44.4)	14 (56.0)	6 (75.0)	0.88 (NS)
Tumor recurrence	39 (41.9)	17 (40.5)	3 (42.9)	1 (50.0)	5 (55.6)	11 (44.0)	2 (25.0)	

Initial therapy								
Upfront surgery[d]								
No								
Yes	77 (83.7)	36 (85.7)	5 (71.4)	2 (100.0)	8 (88.9)	19 (79.2)	7 (87.5)	0.86 (NS)
RT or CRT [b]	15 (16.3)	6 (14.3)	2 (28.6)	0 (0.0)	1 (11.1)	5 (20.8)	1 (12.5)	
RT								
CRT	22 (24.2)	10 (24.4)	1 (14.3)	0 (0.0)	2 (22.2)	8 (33.3)	1 (12.5)	0.93 (NS)
With concomitant 5FU-	61 (67.0)	27 (65.9)	5 (71.4)	2 (100.0)	7 (77.8)	14 (58.3)	6 (75.0)	
MMC	11 (18.0)	5 (18.5)	0 (0.0)	0 (0.0)	2 (28.6)	2 (14.3)	2 (33.3)	
With concomitant 5FU-								
CDDP	47 (77.0)	21 (77.8)	5 (100.0)	2 (100.0)	4 (57.1)	11 (78.6)	4 (66.7)	
With other concomitant								
CT	3 (4.9)	1 (3.7)	0 (0.0)	0 (0.0)	1 (14.3)	1 (7.1)	0 (0.0)	
No RT/CRT	8 (8.8)	4 (9.8)	1 (14.3)	0 (0.0)	0 (0.0)	2 (8.3)	1 (12.5)	
Complete Response after RT/CRT [e]								
Yes	72 (86.7)	34 (91.9)	5 (83.3)	1 (50.0)	7 (77.8)	19 (86.4)	6 (85.7)	0.57 (NS)
No	11 (13.3)	3 (8.1)	1 (16.7)	1 (50.0)	2 (22.2)	3 (13.6)	1 (14.3)	
PIK3CA mutational status								
Wild-type	71 (76.3)	38 (90.5)	4 (57.1)	1 (50.0)	7 (77.8)	16 (64.0)	5 (62.5)	0.08 (NS)
Mutated	22 (23.7)	4 (9.5)	3 (42.9)	1 (50.0)	2 (22.2)	9 (36.0)	3 (37.5)	
HPV viral load								
Low (<12)	41 (44.1)	21 (50.0)	4 (57.1)	1 (50.0)	2 (77.8)	8 (32.0)	5 (62.5)	0.37 (NS)
High (≥12)	52 (55.9)	21 (50.0)	3 (42.9)	1 (50.0)	7 (22.2)	17 (68.0)	3 (37.5)	

[a] Chi-Square Test; *p* values for comparison of the EPI group vs. the 2J-COL group vs. the 2J-NL group vs. the MJ-CL group vs. the MJ-SC group vs. Other group for each parameter

[b] Data available for 91 patients

[c] Data available for 90 patients

[d] Data available for 92 patients

[e] Data available for 83 patients

CDDP: cisplatin; CRT: chemo-radiation; CT: chemotherapy; MMC: mitomycin C; RT: radiation therapy

Supplementary Table 3. Multivariate model including HPV viral load.

Variable		HR	95% CI	p-value
**Tumor stage**	I	1.0		0.10
	II	2.30	0.47-11.2	
	IIIA	2.56	0.19-35.0	
	IIIB	6.87	0.38-126	
	IV	6.99	0.36-135	
**Tumor differentiation**	Well/Moderate	1.0		0.12
	Poor	0.42	0.14-1.25	
**HPV status**	Genotype 16	1.0		0.024
	Other genotypes	5.27	1.24-22.4	
**Concomitant HIV infection**	Yes	1.75	0.54-5.64	0.35
	No	1.0		
**Lymph node invasion**	Yes	0.48	0.12-1.98	0.31

	No	1.0		
**Complete response after RT/CRT**	Yes	0.10	0.00-0.33	**0.0001**
	No	1.0		
**Viral load**	High (≥ 12)	0.35	0.00-0.79	**0.011**
	Low (< 12)	1.0		

Supplementary Table S4. Clinical and biological characteristics of the 93 patients with HPV-positive anal squamous cell carcinoma (ASCC), according to HPV integration in *NFIX* gene.

	**Patients (%)**	**Number of patient (%)**		***p*-value**[a]
		**HPV integration in *NFIX* gene**	**No HPV integration in *NFIX* gene**	
Total	93 (100)	4 (4.3)	89 (95.7)	
Age (years)				
<65	60 (64.5)	3 (75.0)	57 (64.0)	0.93 (NS)
≥65	33 (35.5)	1 (25.0)	32 (36.0)	
Gender				
Male	18 (19.4)	2 (50.0)	16 (18.0)	0.35 (NS)
Female	75 (80.6)	2 (50.0)	73 (82.0)	
Tumor differentiation				
Well/Moderate	83 (89.2)	4 (100.0)	79 (88.8)	0.91 (NS)
Poor	10 (10.8)	0 (0.0)	10 (11.2)	
Basaloid contingent				
Present	9 (9.7)	0 (0.0)	9 (10.1)	0.85 (NS)
Absent	84 (90.3)	4 (100.0)	80 (89.9)	
Integration of HPV				
EPI	42 (45.2)	0 (0.0)	42 (47.2)	**0.036**
2J	15 (16.1)	0 (0.0)	15 (16.9)	
MJ	36 (38.7)	4 (100.0)	32 (36.0)	
HPV status				
Genotype 16	85 (91.4)	4 (100.0)	81 (91.0)	0.78 (NS)
Other genotypes	8 (8.6)	0 (0.0)	8 (8.9)	
Concomitant HIV infection [b]				
Yes	8 (8.8)	0 (0.0)	8 (9.2)	0.79 (NS)
No	83 (91.2)	4 (100.0)	79 (90.8)	
Tumor stage (AJCC 2010) [c]				
I	8 (8.9)	0 (0.0)	8 (9.3)	**0.00016**
II	37 (41.1)	0 (0.0)	37 (43.0)	
IIIA	21 (23.3)	3 (75.0)	18 (20.9)	
IIIB	20 (22.2)	1 (25.0)	19 (22.1)	
IV	4 (4.4)	0 (0.0)	4 (4.7)	
Lymph node invasion [b]				
Yes	40 (44.0)	4 (100.0)	36 (41.4)	0.073 (NS)
No	51 (56.0)	0 (0.0)	51 (58.6)	
Synchronous metastasis [d]				
Yes	4 (4.3)	0 (0.0)	4 (4.5)	0.41 (NS)
No	88 (95.7)	4 (100.0)	84 (95.5)	

Sample status				
Treatment-naive tumor	54 (58.1)	4 (100.0)	50 (56.2)	0.22 (NS)
Tumor recurrence	39 (41.9)	0 (0.0)	39 (43.8)	
Complete Response after RT/CRT [e]				
Yes	72 (86.7)	4 (100.0)	68 (86.1)	0.96 (NS)
No	11 (13.3)	0 (0.0)	11 (13.9)	
PIK3CA mutational status				
Wild-type	71 (76.3)	3 (75.0)	68 (76.4)	0.59 (NS)
Mutated	22 (23.7)	1 (25.0)	21 (23.6)	
HPV viral load				
Low (<12)	41 (44.1)	1 (25.0)	40 (44.9)	0.63 (NS)
High (≥12)	52 (55.9)	3 (75.0)	49 (55.1)	

[a] Chi-Square Test, Chi-Square Test with Yates' correction or Fisher's exact Test, as appropriate; *p* values for comparison of the HPV integration in *NFIX* gene group vs. the No HPV integration in *NFIX* gene group for each parameter

[b] Data available for 91 patients

[c] Data available for 90 patients

[d] Data available for 92 patients

[e] Data available for 83 patients

CRT: chemo-radiotherapy; RT: radiotherapy

Supplementary Figure S1. HPV integration sites in the *NFIX* gene (GRCh37 - hg19).

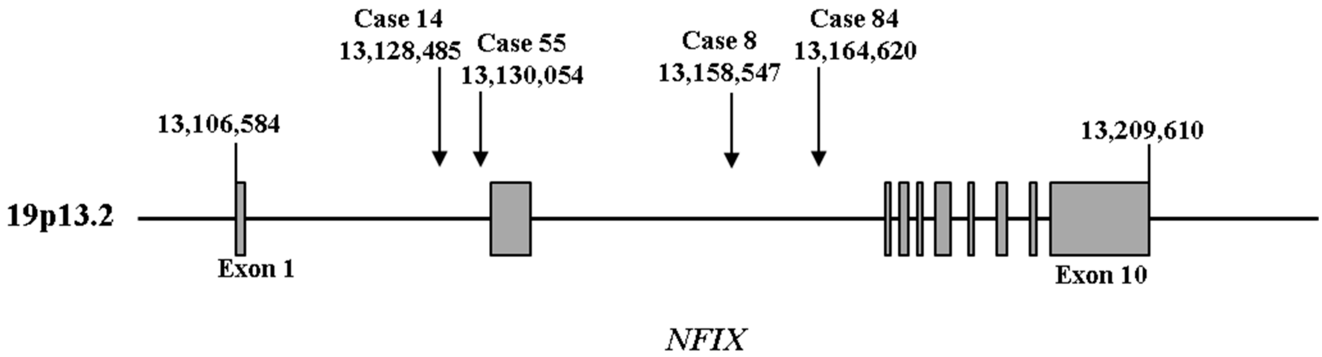

Supplementary Figure 2. Overall survival curves of the 90 patients with HPV-positive anal squamous cell carcinoma (ASCC) evaluable for survival, according to HPV integration signatures. Median OS: 166.2 months in the EPI group vs. 121.6 months the 2J group vs. 92.7 months the MJ group. Log-Rank test, *p*=0.50.

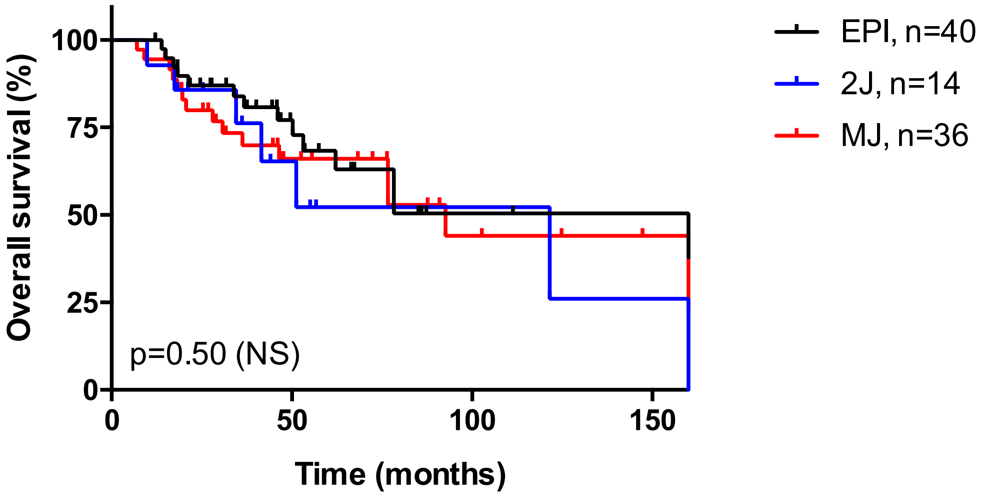

Supplementary Figure 3. Overall survival curves of the patients with HPV-positive anal squamous cell carcinoma (ASCC) evaluable for survival, according to viral load (high: ≥ 12 vs. low: < 12).
a. in the HIV-negative subgroup (n=80). Median OS: 76.7 months in the low viral load group vs. 168.2 months in the high viral load group. Log-Rank test, hazard ratio (HR): 2.04, *p*=0.038.

b. in the radiotherapy (RT) subgroup (n=22). Median OS: 27.9 months in the low viral load group vs. 220.4 months in the high viral load group. Log-Rank test, hazard ratio (HR): 6.30, *p*=0.0006.

c. in the chemoradiotherapy (CRT) subgroup (n=61). Median OS: 62.2 months in the low viral load group vs. not reached in the high viral load group. Log-Rank test, hazard ratio (HR): 1.29, *p*=0.54.

d. in the complete response (CR) subgroup (n=72). Median OS: 62.2 months in the low viral load group vs. 220.4 months in the high viral load group. Log-Rank test, hazard ratio (HR): 3.09, *p*=0.0022.

e. in the non-CR subgroup (n=11). Median OS: 18.4 months in the low viral load group vs. 25.8 months in the high viral load group. Log-Rank test, hazard ratio (HR): 2.38, *p*=0.25.

a.

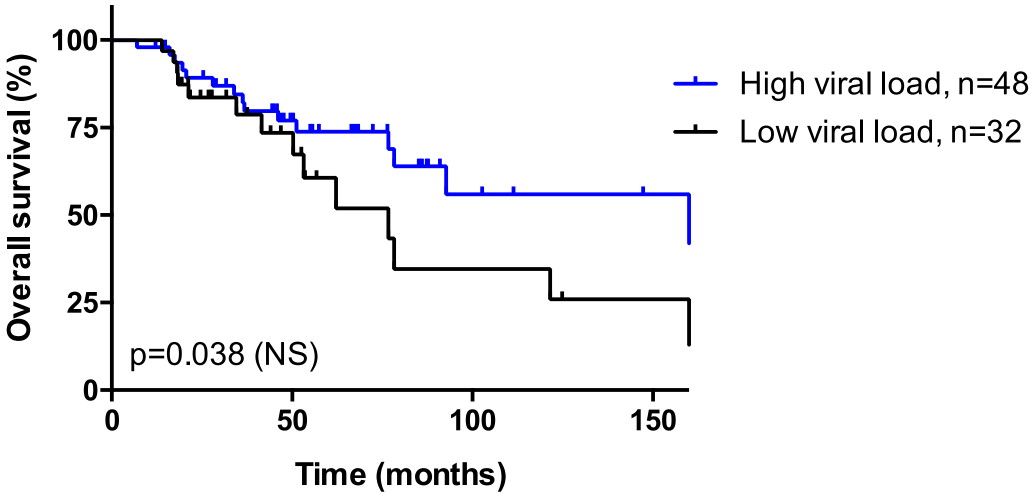

b.

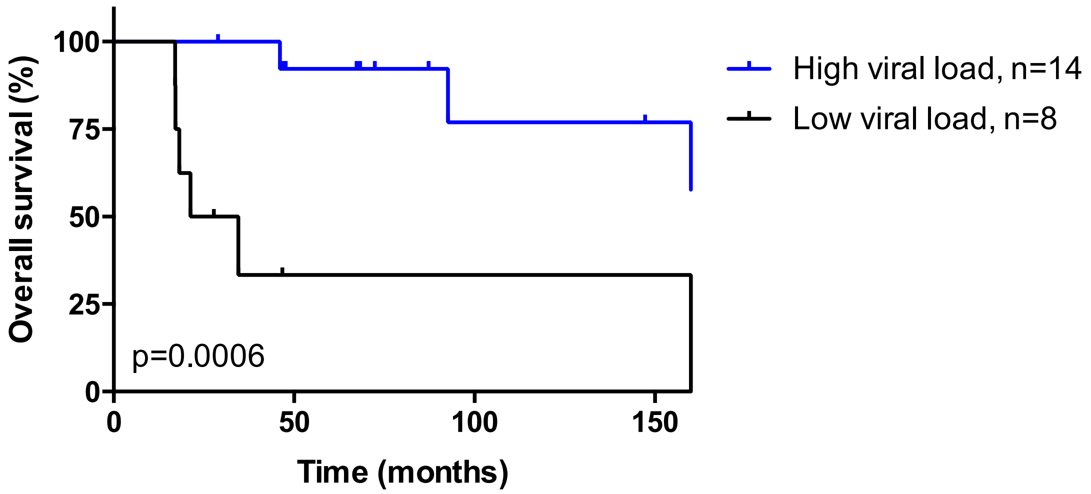
Overall survival (%)
100
75
50
25
0
p=0.0006
0
50
100
150
Time (months)
High viral load, n=14
Low viral load, n=8

c.

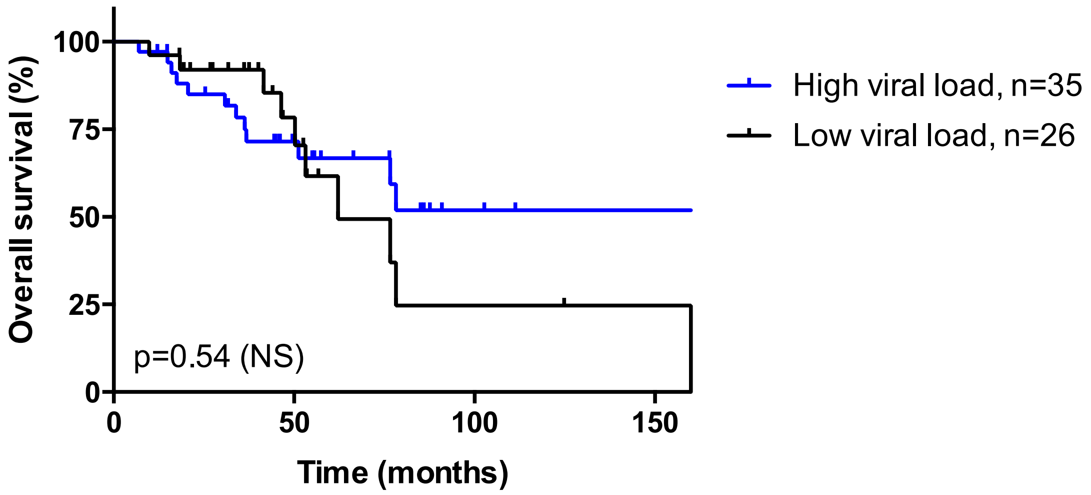
Overall survival (%)
100
75
50
25
0
p=0.54 (NS)
0
50
100
150
Time (months)
High viral load, n=35
Low viral load, n=26

d.

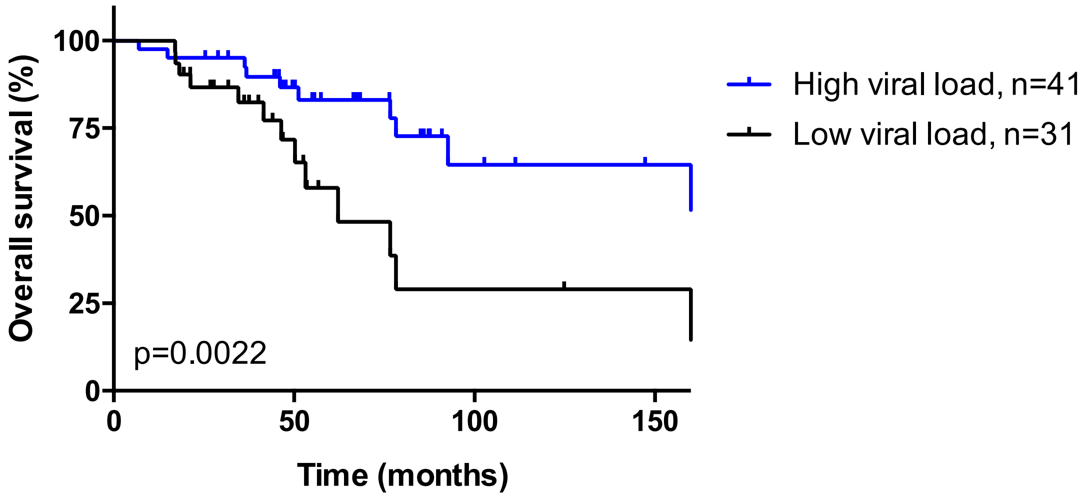
Overall survival (%)
100
75
50
25
0
p=0.0022
0
50
100
150
Time (months)
High viral load, n=41
Low viral load, n=31

e.

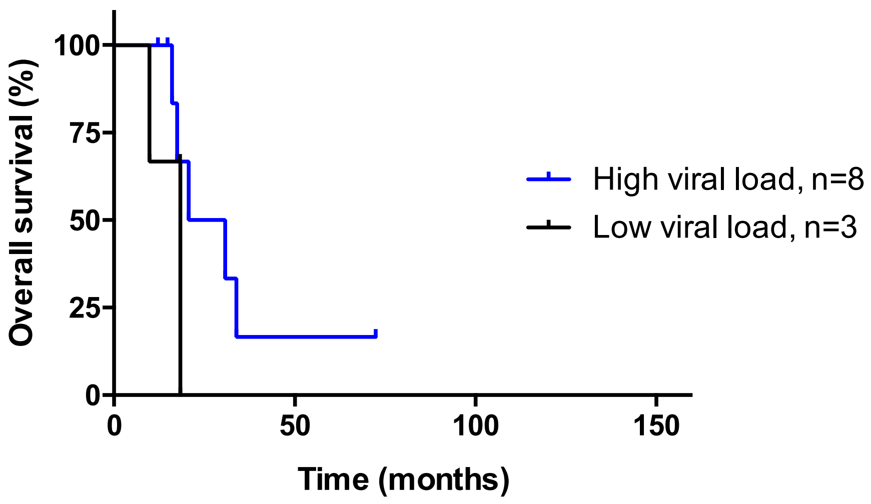
Overall survival (%)
100
75
50
25
0
0
50
100
150
Time (months)
High viral load, n=8
Low viral load, n=3
